# A Genomic-Clinicopathologic Nomogram for the Prediction of Lymph Node Invasion in Prostate Cancer

**DOI:** 10.1155/2021/5554708

**Published:** 2021-05-26

**Authors:** Zongtai Zheng, Shiyu Mao, Zhuoran Gu, Ruiliang Wang, Yadong Guo, Wentao Zhang, Xudong Yao

**Affiliations:** Department of Urology, Shanghai Tenth People′s Hospital, Tongji University School of Medicine, Shanghai, China

## Abstract

**Background:**

Lymph node status is important for treatment decision making in prostate cancer (PCa). We aimed to develop a genomic-clinicopathologic nomogram for the prediction of lymph node invasion (LNI) in PCa.

**Methods:**

Differentially expressed genes between LNI and non-LNI PCa samples were identified in the Cancer Genome Atlas database. Univariate Cox regression analysis and minimum redundancy maximum relevance were performed for gene selection. The synthetic minority oversampling technique (SMOTE) was conducted to balance the minority group (LNI group). Machine learning models were constructed in the training set and assessed in the validation set. Univariable logistic regression and multivariable logistic regression were applied to build a nomogram. Furthermore, the RNA-sequence data from our center were used to validate the expression levels of hub genes between five matched primary PCa and the corresponding LNI samples.

**Results:**

The 37-gene-based support vector machine (SVM) model had the optimal synthesized performance in the SMOTE-balanced training (area under the curve (AUC): 0.947) and validation (AUC: 0.901) sets. Incorporating the SVM-based risk score and the Gleason grade, the genomic-clinicopathologic nomogram demonstrated good prediction and calibration both in the SMOTE-balanced training (AUC: 0.946) and validation (AUC: 0.910) sets. The dysregulated expression of hub genes between PCa and LNI samples was also validated.

**Conclusion:**

The proposed nomogram combining the 37-gene-based SVM model with the Gleason grade had the potential to preoperatively predict LNI in PCa. Some of the hub genes should be prioritized for functional studies and mechanistic analyses.

## 1. Introduction

Prostate cancer (PCa) is the most common cancer in men with a rising global disease burden on public health [1]. Up to 15% of PCa patients present lymph node invasion (LNI) which is a negative prognostic factor [2]. Accurate nodal staging is important for identifying PCa patients who may benefit from additional treatment [3, 4]. Extended pelvic lymph node dissection (ePLND) is the gold standard for nodal staging [5]. However, due to the increased risk of potential morbidity and prolonged operative time [6, 7], the current European Association of Urology guidelines recommend that radical prostatectomy (RP) combined with an ePLND is only performed in PCa patients with the estimated risk for LNI >5% [5, 8].

Currently, there are some nomograms built for the prediction of LNI in PCa, but the predictive factors of these nomograms highly rely on clinical parameters and biopsy reports, and some of the clinical parameters may not be routinely available in other institutions [8–12]. The use of medical imaging techniques for preoperative nodal staging is not recommended due to the low sensitivity [11, 13].

Sequencing of RNA using next-generation sequencing technology (RNA-sequence) is an efficient approach to investigate the difference of PCa in terms of lymph node status at a genomic level. The analysis strategy for RNA-sequence data has evolved from single gene analysis to develop machine learning models based on a set of genes [13, 14], which may be a useful way to innovate clinical management.

Notably, due to the relatively low proportion of LNI PCa patients, data were imbalanced between LNI patients and non-LNI patients. Machine learning models based on imbalanced data are more inclined to ignoring the minority type and representing the majority type, resulting in a bias in performance [15]. The synthetic minority oversampling technique (SMOTE) is a popular and powerful method for balancing data via synthetic data [16]. It generates new minority samples based on available minority data. After data balancing, imbalanced types could be avoided and the performance of prediction models could be effectively improved [15].

In our study, we sought to use the RNA-sequence data from the Cancer Genome Atlas (TCGA) to construct machine learning models for the prediction of LNI in PCa. Simultaneously, the prediction power of machine learning models with and without data balancing was further evaluated. The optimal prediction model was further integrated with clinicopathological features to develop a genomic-clinicopathologic nomogram for predicting LNI.

## 2. Materials and Methods

### 2.1. Ethics

The study was reviewed and approved by the Ethics Committee of Shanghai Tenth People's Hospital (approval number: SHSY-IEC-2014RES-36) and conducted following the ethical standards.

### 2.2. Data Collection

Five primary PCa samples and corresponding LNI samples were obtained from PCa patients that underwent RP and ePLND in Shanghai Tenth People's Hospital. These five patients had a prostate-specific antigen (PSA) level >10 ng/mL or a Gleason grade >6 and a tumor stage of at least cT2. Supplementary Table 2 presents the clinicopathological characteristics of these patients. The ePLND template includes the obturator, external iliac, internal iliac, common iliac, and presacral regions (nine fields) bilaterally, and the margins of the ePLND include the following: the caudal margin was the femoral canal and the deep circumflex vein, the cranial margin was the ureter crossing over the common iliac artery, the lateral margin was the genitofemoral nerve, and the medial margin was the vesical fat [17]. Informed consent was prior obtained from patients. Tumor tissues from the same histologic component of both the primary PCa samples and LNI samples were macrodissected by a genitourinary pathologist (over 10 years of experience) from formalin-fixed paraffin embedded (FFPE) sections, and RNA was isolated. Total RNA was isolated using the QubitRNA Assay Kit (cat. Q32852; Life Technologies, USA) following the manufacturer's protocol. RNA-seq and data analysis were performed by Oebiotech (Shanghai OEbiotech Co., Ltd., Shanghai, China).

The mRNA (RNA-sequence) Fragments Per Kilobase of transcript per Million Fragments standardized expression dataset and corresponding clinicopathological features were downloaded for 426 PCa patients from the Cancer Genome Atlas (TCGA) (http://cancergenome.nih.gov/). Patients without information on lymph node status were excluded from the analysis.

### 2.3. Hub Gene Screening

With the cutoff criterion of |log2 fold change (FC)|>0.5 and FDR (false discovery rate) < 0.05, differentially expressed genes (DEGs) between LNI and non-LNI samples were selected. Then, DEGs were introduced into univariate Cox regression analysis to screen for DFS- (disease-free-survival-) associated genes.

### 2.4. Prediction Model Construction

The ratio of LNI patients to non-LNI patients was 1 : 4.4 in TCGA, indicating a sample imbalance. Therefore, the SMOTE algorithm was was used to balance the minority class. Based on the expression of DFS-associated genes, the PCa patients were equalized via the SMOTE, so that the two classes of PCa patients were 1 : 1 (347 LNI patients and 347 non-LNI patients). Subsequently, PCa patients were randomly divided into the training set and validation set at a ratio of 7 : 3 in imbalanced and SMOTE-balanced datasets, respectively.

DFS-associated genes were conducted using the minimum redundancy maximum relevance (mRMR) algorithm for gene ranking via mutual information (MI) in imbalanced and SMOTE-balanced datasets, respectively. The mRMR algorithm is a supervised feature selection model which initially calculates the MI between features and a target variable. It ranks the features via maximizing MI to the target variable and then minimizes the average MI for features with higher rankings [18]. In this way, the top 40 genes were selected for developing machine learning models, including support vector machine (SVM) and least absolute shrinkage and selection operator (LASSO) models.

A nonlinear SVM-based recursive feature elimination (SVM-RFE) algorithm was applied to investigate the optimal number of genes and obtain the most relevant genes for SVM model construction. This algorithm included backward elimination in each iteration, wherein features that minimally improve the performance of the model were removed [19].

The LASSO algorithm removes the genes that minimally influence the target variable and selects the genes with nonzero coefficients for model construction. The risk score of the LASSO model was calculated by summing the selected genes weighted by their coefficients.

The two models were developed via 10-fold cross-validation in the training set to obtain the optimal parameter configuration for each model and were then assessed in the validation set. The area under the receiver operator characteristic (ROC) curve (AUC) was used to evaluate the performance of each model. Accuracy, sensitivity, specificity, negative predictive value (NPV), and positive predictive value (PPV) were calculated according to the Youden index [20]. The machine learning model with the highest accuracy, sensitivity, and AUC was selected for nomogram construction. The median value of the machine learning model-based risk score was used to divide patients into low- and high-risk groups in the training and validation sets, respectively.

### 2.5. Nomogram Development

We selected the primary PCa patients with clinical characteristics in the SMOTE-balanced training set to develop a nomogram. The risk score generated by the optimal machine learning model and preoperative clinical characteristics, including age, PSA level (most recent), clinical stage, and Gleason grade, were introduced into the univariate logistic analysis. Significant factors in the univariate logistic analysis were put into the step-wise multivariate logistic regression analysis. A forward stepwise selection was used with Akaike's information criterion (AIC) as the stopping rule. Variance inflation factors (VIFs) were calculated to evaluate the collinearity of the multivariate logistic regression. A nomogram was constructed using the coefficients of factors chosen by multivariate logistic regression.

AUC and calibration curves were applied to investigate the diagnostic power of the nomogram. Decision curve analysis (DCA) was applied to investigate the clinical utility of the nomogram for decision making. The Hosmer–Lemeshow test and Harrell's concordance index (C-index) were performed to quantify the performance of the nomogram. Furthermore, we calculated the net reclassification improvement (NRI) and the integrated discrimination improvement (IDI) to evaluate the incremental prediction ability of the nomogram compared with the Gleason grade [21].

### 2.6. Gene Set Enrichment Analysis (GSEA)

GSEA (http://www.broadinstitute.org/gsea/index.jsp) was conducted to investigate signaling pathways between PCa patients in high- and low-risk groups. Signaling pathways with *P* < 0.05 and a false discovery rate <0.25 were considered statistically significant.

### 2.7. Statistical Analysis

Statistical analysis was conducted with R statistical software (version 3.6.1 R, https://www.r-project.org/). Supplementary Table 1 presents the R packages used for statistical analysis. The clinical characteristics between the training and validation sets were compared using Student's *t*-test, the chi-square test, or the Mann–Whitney *U* test, as appropriate. The differences in the expression levels of hub genes between PCa samples and LNI samples were compared using the Wilcoxon test. All machine learning models were constructed on the training set and assessed on the validation set. All tests were 2-tailed, and *P* value < 0.05 was regarded as statistically significant.

## 3. Results

### 3.1. Patient Population

**Figure 1:**
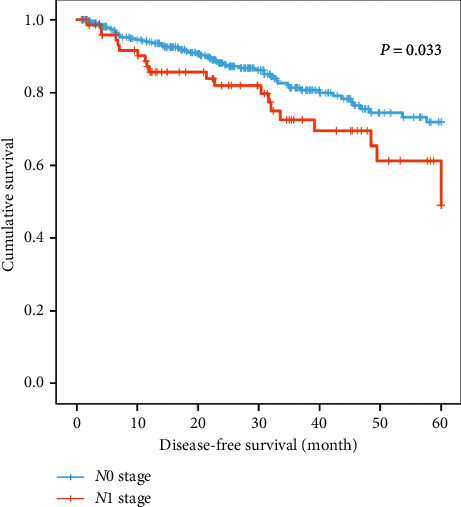
Kaplan–Meier curves of disease-free survival for prostate cancer patients based on the lymph node status.

A total of 426 PCa patients (347 LNI patients and 79 non-LNI patients) were obtained from TCGA database. Survival analysis revealed that LNI patients had poorer DFS than non-LNI patients within five years (*P* = 0.033, Figure 1).

### 3.2. Hub Gene Screening and Prediction Model Development

A total of 1538 DEGs (546 upregulated and 992 downregulated) were screened from TCGA by univariate Cox regression analysis. As a result, 314 DFS-associated genes were determined. The top 40 genes ranked by the mRMR algorithm were retained for prediction model construction in imbalanced and SMOTE-balanced training sets, respectively.

**Figure 2:**
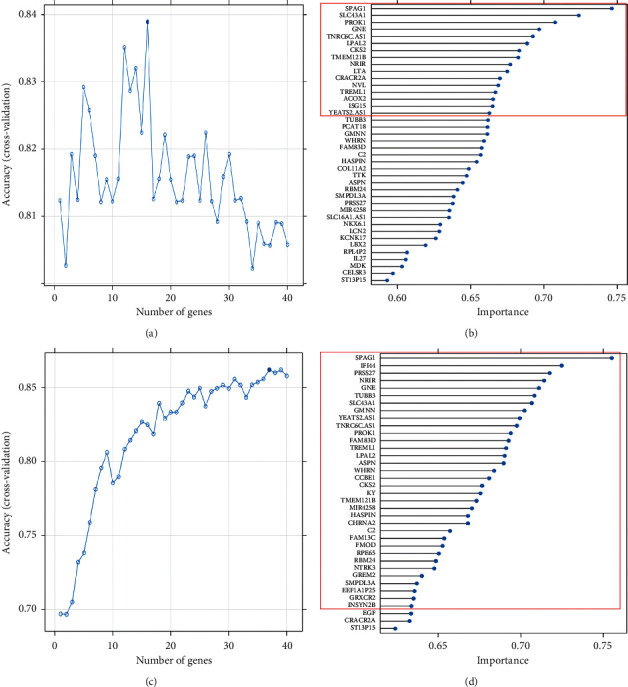
Construction of SVM models. (a) Gene selection process using SVM-RFE and 10-fold cross-validation in the imbalanced training set: 16 genes with the highest discriminative accuracy were selected for SVM model development. (b) SVM-RFE is used to rank genes according to the gene importance, and the top 16 genes were selected for SVM model development. (c) Gene selection process using SVM-RFE and 10-fold cross-validation in the SMOTE-balanced training set: 37 genes with the highest discriminative accuracy were selected for SVM model development. (d) SVM-RFE is used to rank genes according to the gene importance, and the top 37 genes were selected for SVM model development. SVM: support vector machine; SVM-RFE: SVM-based recursive feature elimination; SMOTE: synthetic minority oversampling technique.

**Figure 3:**
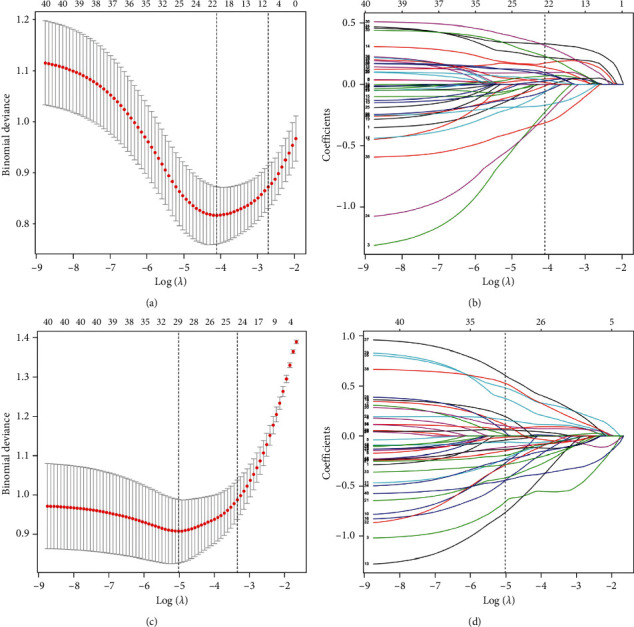
Construction of LASSO models. (a) Selection of the tuning parameter *λ* in the LASSO model via 10-fold cross-validation in the imbalanced training set. The optimal *λ* value of 0.0165, with log (*λ*) = −4.103, was chosen based on minimum criteria. (b) LASSO coefficient profiles of the 22 genes. (c) Selection of the tuning parameter *λ* in the LASSO model via 10-fold cross-validation in the SMOTE-balanced training set. The optimal *λ* value of 0.0066, with log (*λ*) = −5.021, was chosen based on minimum criteria. (d) LASSO coefficient profiles of the 29 genes. LASSO: least absolute shrinkage and selection operator; SMOTE: synthetic minority oversampling technique.

Sixteen and 37 genes chosen by the SVM-RFE algorithm were conducted to construct SVM models with the highest accuracy in imbalanced and SMOTE-balanced training sets, respectively (Figure 2). Twenty-two and 29 genes with nonzero coefficients were selected by the LASSO algorithm to develop LASSO models with the least binominal deviance in imbalanced and SMOTE-balanced training sets, respectively (Figure 3).

### 3.3. Performance of Machine Learning Models

**Figure 4:**
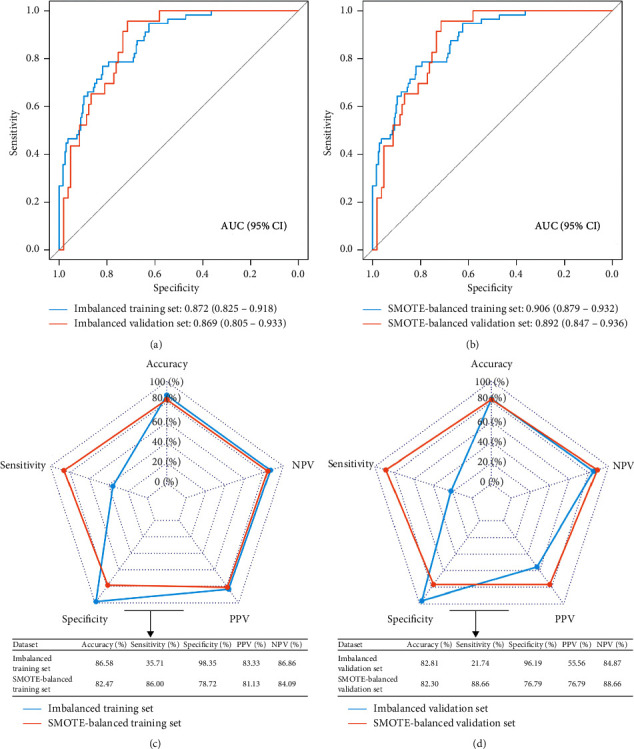
Performance of LASSO models in imbalanced and SMOTE-balanced datasets. (a) ROC curves of the LASSO model in imbalanced training and validation sets. (b) ROC curves of the LASSO model in SMOTE-balanced training and validation sets. (c) The predictive performance of LASSO models in imbalanced and SMOTE-balanced training sets. (d) The predictive performance of SVM models in imbalanced and SMOTE-balanced validation sets. ROC: receiver operating curve; AUC: area under the ROC curve; NPV: negative predictive value; PPV: positive predictive value; LASSO: least absolute shrinkage and selection operator; SMOTE: synthetic minority oversampling technique.

**Figure 5:**
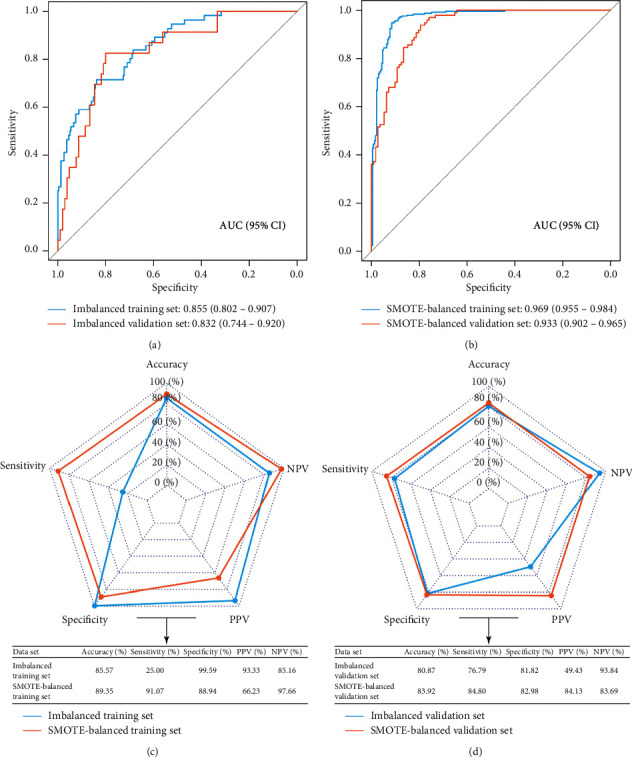
Performance of SVM models in imbalanced and SMOTE-balanced datasets. (a) ROC curves of the SVM model in imbalanced training and validation sets. (b) ROC curves of the SVM model in SMOTE-balanced training and validation sets. (c) The predictive performance of SVM models in imbalanced and SMOTE-balanced training sets. (d) The predictive performance of SVM models in imbalanced and SMOTE-balanced validation sets. ROC: receiver operating curve; AUC: area under the ROC curve; NPV: negative predictive value; PPV: positive predictive value; SVM: support vector machine; SMOTE: synthetic minority oversampling technique.

As for LASSO models, we observed high accuracy and specificity but low sensitivity and relatively low AUC in the imbalanced dataset (Figure 4). In contrast, although the accuracy and specificity of the LASSO model declined in the SMOTE-balanced dataset, the sensitivity improved greatly, with the sensitivity increasing from 35.71% to 86% and from 21.74% to 88.66% in SMOTE-balanced training and validation sets, respectively (Figures 4(c) and 4(d)). The specificity, PPV, and NPV of SVM models in imbalanced and SMOTE-balanced datasets were relatively equal, and the AUC, accuracy, and sensitivity of the 37-gene-based SVM model in the SMOTE-balanced dataset were higher than those of the 16-gene-based SVM model in the imbalanced dataset (Figure 5).

Furthermore, the synthesized performance of the 37-gene-based SVM model in the SMOTE-balanced dataset was better than that of LASSO models both in imbalanced and SMOTE-balanced datasets. In this way, the 37-gene-based SVM model and the risk score of each PCa patient generated by the 37-gene-based SVM model were selected for further analysis.

**Figure 6:**
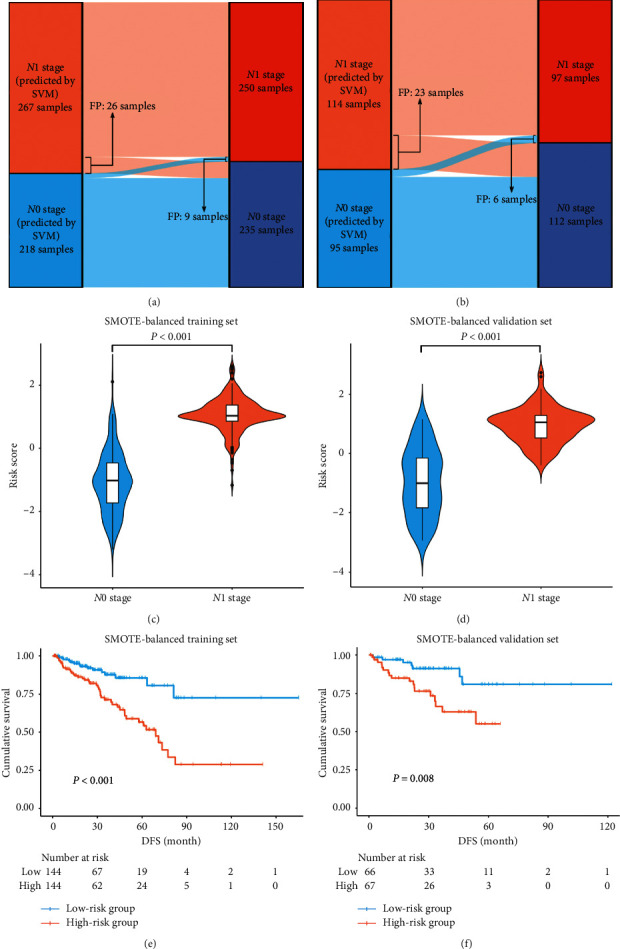
Performance of the SVM-based risk score in the SMOTE-balanced dataset. Alluvial diagrams showed the predictive results of the 37-gene-based SVM model in SMOTE-balanced training (a) and validation (b) sets. Violin plots of the risk score in SMOTE-balanced training (c) and validation (d) sets grouped by the N stage. Kaplan–Meier curves of DFS for prostate cancer patients based on the risk group in SMOTE-balanced training (e) and validation (f) sets. DFS: disease-free survival; FP: false positive; FN: false negative; SVM: support vector machine; SMOTE: synthetic minority oversampling technique.

The Pearson correlation coefficients among the 37 genes in the 37-gene-based SVM model were all <0.7, indicating no collinearity between genes. Alluvial diagrams presented the predictive results of the 37-gene-based SVM model in the SMOTE-balanced training and validation sets (Figures 6(a) and 6(b)). LNI patients had significantly higher risk scores than non-LNI patients both in the SMOTE-balanced training and validation sets (both *P* < 0.001, Figures 6(c) and 6(d)).

### 3.4. The Prognostic Value and Pathway Analysis of the SVM Model

PCa patients in the high-risk group had poorer DFS than PCa patients in the low-risk group in the SMOTE-balanced training and validation sets (Figures 6(e) and 6(f)).

**Figure 7:**
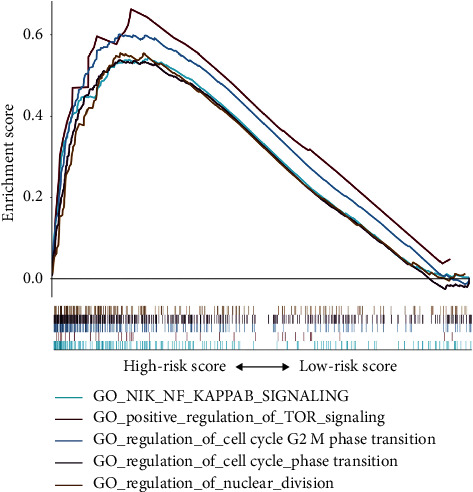
Gene set enrichment analysis revealed that the high-risk group was enriched for hallmarks of malignant tumors.

GSEA revealed that malignant hallmarks of tumors, including “NIK_NF_KAPPAB_SIGNALING,” “POSITIVE_REGULATION_OF_TOR_SIGNALING,” “REGULATION_OF_CELL_CYCLE_G2_M_PHASE_TRANSITION,” “REGULATION_OF_CELL_CYCLE_PHASE_TRANSITION,” and “REGULATION_OF_NUCLEAR_DIVISION,” were mainly enriched in the high-risk group (Figure 7).

### 3.5. Development and Performance of the Genomic-Clinicopathologic Nomogram

After removing the synthetic samples generated by the SMOTE, there were 291 and 135 primary PCa patients with clinical characteristics in SMOTE-balanced training and validation sets, respectively.  Table 1 presents the clinical characteristics of PCa patients. No significant differences were observed in age, PSA level (most recent), clinical stage, pathologic stage, Gleason grade, surgical margin resection status, number of dissected lymph nodes, number of positive lymph nodes, and N stage between the two datasets. After univariate and multivariate analyses, the risk score and Gleason grade remained significant factors for the prediction of LNI with the lowest AIC value (AIC = 136.5) (Table 2). The VIFs of risk score and Gleason grade were 1.509 and 1.611, respectively, indicating no collinearity.

**Figure 8:**
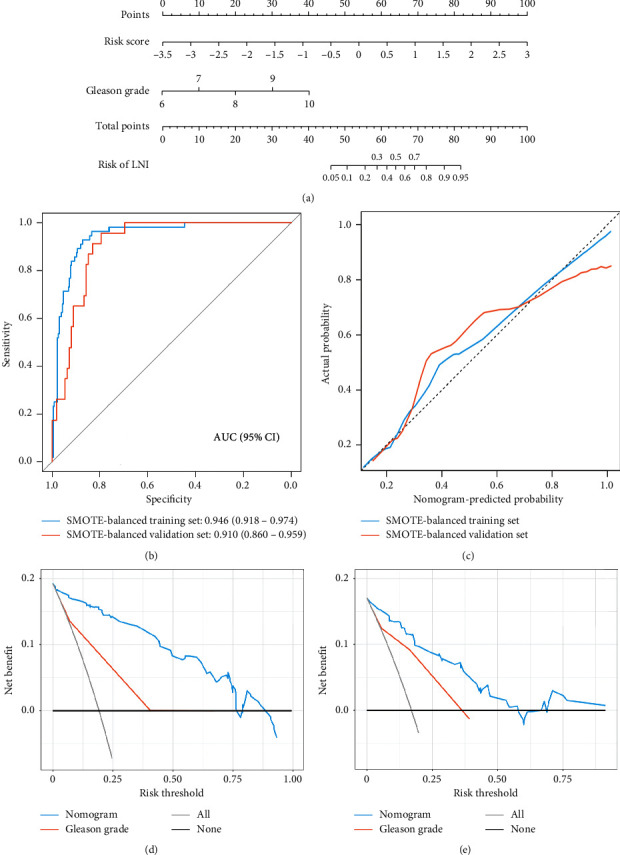
Construction and performance of the genomic-clinicopathologic nomogram. (a) Nomogram to predict the LNI. (b) ROC curves of the nomogram in SMOTE-balanced training and validation sets after removing the synthetic samples generated by the SMOTE. (c) Calibration curve of the nomogram in SMOTE-balanced training and validation sets. DCA for risk score and nomogram in SMOTE-balanced training (d) and validation (e) sets. ROC: receiver operating curve; AUC: area under the ROC curve; LNI: lymph node invasion; SMOTE: synthetic minority oversampling technique.

Then, the risk score and Gleason grade were used to construct a genomic-clinicopathologic nomogram (Figure 8(a)). After removing the synthetic samples generated by the SMOTE, the AUCs of the nomogram were 0.946 (95% confidence interval (CI): 0.918–0.974) and 0.910 (95% CI: 0.860–0.959) in SMOTE-balanced training and validation sets, respectively (Figure 8(b)). The calibration curves showed marked calibration of the prediction and observation in both datasets (Figure 8(c)). Harrell's C-indices of the nomogram were 0.946 (95% CI, 0.918–0.974) and 0.910 (95% CI, 0.861–0.958) in SMOTE-balanced training and validation sets, respectively. The Hosmer–Lemeshow test yielded nonsignificant *P* values of 0.749 and 0.846 in SMOTE-balanced training and validation sets, respectively, indicating good calibration power. The DCA showed that the nomogram had a higher clinical net benefit than the Gleason grade in both datasets (Figures 8(d) and 8(e)). Compared with the Gleason grade, the nomogram significantly improved diagnostic accuracy (overall category-based NRI, 0.23; NRI indices for events and nonevents, 7.59% and 14.99%, respectively; IDI, 0.32, all *P* < 0.001), and similar results were also observed in SMOTE-balanced training and validation sets, respectively, which are presented in Table 3.

### 3.6. Validation of 37 Genes from the SVM Model

**Figure 9:**
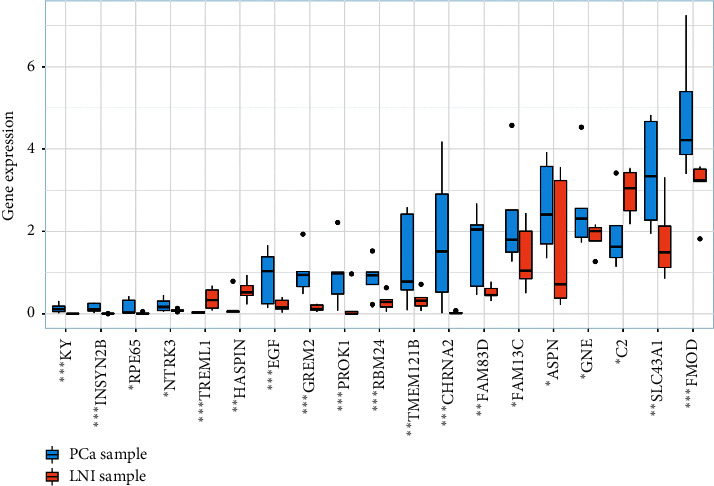
Dysregulated expression of 18 genes between five matched primary PCa and corresponding LNI samples using the RNA-sequence data in our center. PCa: prostate cancer; LNI: lymph node invasion.  ^*∗*^*P* < 0.05;  ^*∗*^ ^*∗*^*P* < 0.01;  ^*∗*^ ^*∗*^ ^*∗*^*P* < 0.001.

The RNA-sequence data of five primary PCa samples and corresponding LNI samples from our center were used to validate the expression levels of 37 genes from the SVM model. The results demonstrated that the expression levels of 18 genes were significantly different between PCa and LNI samples (Figure 9).

## 4. Discussion

The lymph node status is essential for decision making regarding PCa treatment regimens, specifically concerning the use of ePLND and additional therapies [3, 4]. Current guidelines for PCa patients with LNI suggest that ePLND should be a necessary part of RP [22], but this procedure extends the operative time and increases the risk of potential morbidity [6, 7]. Therefore, accurate nodal staging could reduce unnecessary ePLND.

In this study, we constructed genomic-based machine learning models for the prediction of LNI. However, due to the relatively low proportion of LNI PCa patients, data were imbalanced between the two types (LNI patients vs. non-LNI patients, 1 : 4.4). The synthesized performance of machine learning models based on the imbalanced dataset was unsatisfactory, with indeed low sensitivity. After data balancing by SMOTE, these machine learning models achieved better synthesized performance, suggesting that it is useful to develop prediction models with SMOTE-balanced data. As the 37-gene-based SVM model built using the SMOTE-balanced training data had the optimal synthesized performance in the prediction of LNI, the risk score generated by this model was selected for the nomogram construction. A genomic-clinicopathologic nomogram combined with the risk score and Gleason grade achieved novel calibration and good clinical net benefit, indicating a useful approach for the preoperative prediction of LNI.

Currently, medical imaging techniques, including CT, MRI, and PET, have been commonly used for preoperative N staging in PCa. However, these methods rely on experienced radiologists, which could inevitably result in human error and low sensitivity, as metastatic lymph nodes may have normal size [23, 24]. Medical image-based radiomics has been used for preoperative N staging in PCa cancer imaging. However, the cohorts of these studies were relatively small [25, 26], and the application of radiomics is still hindered by multiple reasons, including lack of standardization, automation, and harmonization.

At present, several nomograms have been constructed for predicting LNI based on biopsy data [8–12]. Briganti et al. [9] built a nomogram predicting the risk of LNI in PCa patients undergoing RP combined with ePLND. The nomogram contains the routinely available clinical factors such as clinical stage, preoperative PSA, and biopsy Gleason sum with relatively high accuracy. Then, Briganti et al. [10] further found that the inclusion of the percentage of positive biopsy cores could improve the performance of the nomogram. In 2012, Briganti et al. updated the nomogram in more contemporary patients undergoing RP combined with ePLND and reported that patients with an LNI risk <5% might be safely spared ePLND based on the nomogram [8]. Gandaglia et al. [11] constructed a nomogram based on detailed biopsy reports including the percentage of positive cores with highest-grade PCa and the percentage of positive cores with lower-grade PCa. Then, Gandaglia et al. [12] developed the first nomogram including mpMRI and MRI-targeted biopsy data. Adoption of this nomogram could avoid up to 60% of ePLND at the cost of missing only 1.6% of LNI patients. However, all of the above models highly rely on the clinical factors and biopsy reports without the application of genomic data.

With the development of high-throughput sequencing technologies, a genomic signature may be a useful tool for predicting LNI. Cao et al. [27] used the clinical and RNA-sequence data of PCa patients from TCGA to develop a nomogram based on 7-gene risk signature, PSA, clinical stage, and primary and secondary biopsy Gleason grade for N staging, with the AUC of 0.902%. However, there was no validation set in this study, and the sample imbalance was not equalized. In our study, the genomic-clinicopathologic nomogram integrating the SVM-based risk score with the Gleason grade achieved favorable performance in the prediction of LNI, with the AUCs of 0.946 and 0.910 in SMOTE-balanced training and validation sets, respectively. The predictive factors of this genomic-clinicopathologic nomogram could be obtained from biopsy tissues before RP. Hence, the proposed nomogram may be useful in the prediction of LNI and the preoperative selection of ePLND candidates.

The results of the RNA sequence in our center showed that 18 of 37 genes from the SVM model exhibited dysregulated expression between PCa and LNI samples, indicating that dysregulated expression levels of these genes played an important role in the LNI of PCa.

This study had some limitations. The nomogram was not validated in an external validation from different institutions. Multicenter studies are needed to evaluate the generalizability of the proposed nomogram. In addition, the number of tumor samples for RNA sequence in our center was small.

In conclusion, a genomic-clinicopathologic nomogram integrating the SVM-based risk score with the Gleason grade had encouraging performance in the preoperative prediction of LNI and may provide added value for the preoperative selection of ePLND candidates in PCa. Dysregulated expression of genes from the SVM model between PCa and LNI samples was validated by RNA sequence in our center.

## Figures and Tables

**Table 1:** The clinicopathological characteristics of primary patients in SMOTE-balanced training and validation sets.

	Number of patients (%)		*P* value
Characteristic	SMOTE-balanced training set (*n* = 291)	SMOTE-balanced validation set (*n* = 135)	
Age, years			
Mean [median]	61.50 [62]	61.15 [62]	0.640^a^
IQR	57–66	56–66	

PSA level (most recent), ng/mL			
Mean [median]	1.17 [0.10]	1.00 [0.10]	0.698^a^
IQR	0.03–0.18	0.03–0.17	

Clinical stage			
cT1	93 (31.96)	44 (32.59)	0.294^b^
cT2	104 (35.74)	55 (40.74)	
cT3	30 (10.31)	19 (14.07)	
cT4	1 (0.34)	0 (0)	
NA	63 (21.65)	17 (12.59)	

Pathologic stage			
pT2	98 (33.68)	47 (34.81)	0.534^b^
pT3	181 (62.20)	85 (62.96)	
pT4	10 (3.44)	1 (0.74)	
NA	2 (0.69)	2 (1.48)	

Gleason grade			
<7	17 (5.84)	7 (5.19)	0.739^b^
= 7	140 (48.11)	69 (51.11)	
>7	134 (46.05)	59 (43.70)	

Surgical margin resection status			
R0	178 (61.17)	89 (65.93)	0.283^b^
R1	97 (33.33)	36 (26.67)	
R2	2 (0.69)	2 (1.48)	
NA	14 (4.81)	8 (5.93)	

Number of dissected lymph nodes			
Mean [median]	12.15 [10]	10.75 [9]	0.170^a^
IQR	5–16	5–15	

Number of positive lymph nodes			
Mean [median]	0.51 [0]	0.35 [0]	0.279^a^
IQR	0	0	

*N* stage			
*N*0	235 (80.76)	112 (38.49)	0.297^c^
*N*1	56 (19.24)	23 (7.90)	

IQR: interquartile range; NA: not available; PSA: prostate-specific antigen; SMOTE: synthetic minority oversampling technique; ^a^*t*-test; ^b^Mann–Whitney *U* test; ^c^chi-square test.

**Table 2:** Univariate and multivariate logistic analyses in the SMOTE-balanced training set.

Variable	Univariate analysis			Multivariate analysis		
	*β*	OR (95% CI)	*P* value	*β*	OR (95% CI)	*P* value
Age (continuous), years	0.004	1.004 (0.961–1.049)	0.868	—	—	—
PSA level (most recent)	0.021	1.021 (0.959–1.086)	0.516	—	—	—
Clinical stage	0.501	1.650 (1.041–2.615)	0.033	—	—	—
Gleason grade	1.098	2.997 (2.102–4.272)	<0.001	1.071	2.919 (1.962–4.343)	<0.001
Risk score	2.590	13.334 (6.888–25.815)	<0.001	2.539	12.670 (6.211–25.847)	<0.001

CI: confidence interval; OR, odds ratio; PSA: prostate-specific antigen; SMOTE: synthetic minority oversampling technique.

**Table 3:** The NRI and IDI indices.

	Primary dataset		SMOTE-balanced training set		SMOTE-balanced validation set	
	Values	*P* value	Values	*P* value	Values	*P* value
NRI	0.23	<0.001	0.25	<0.001	0.58	<0.001
Events NRI	7.59%		8.93%		56.52%	
Nonevents NRI	14.99%		16.17%		1.79%	
IDI	0.32	<0.001	0.39	<0.001	0.18	<0.001

NRI: net reclassification improvement; IDI: integrated discrimination improvement; SMOTE: synthetic minority oversampling technique.

## Data Availability

The RNA-sequence data and corresponding clinicopathological features were retrieved from TCGA (http://cancergenome.nih.gov/), which are openly available.

## Supplementary Materials

Supplementary Materials Supplementary Figure 1: pairwise Spearman rank correlation among 37 selected genes of the support vector machine model in the SMOTE-balanced training set. Supplementary Table 1: R packages used in this study. Supplementary Table 2: the clinicopathological characteristics of five prostate cancer patients in Shanghai Tenth People's Hospital.
