# Supplementary material for: A Genomic-Clinicopathologic Nomogram for the Prediction of Lymph Node Invasion in Prostate Cancer
Source: J Oncol. 2021 May 26;2021:5554708. doi: 10.1155/2021/5554708 (PMC8172299; doi:10.1155/2021/5554708)
Supplement: Supplementary Materials — Supplementary Figure 1: pairwise Spearman rank correlation among 37 selected genes of the support vector machine model in the SMOTE-balanced training set. Supplementary Table 1: R packages used in this study. Supplementary Table 2: the clinicopathological characteristics of five prostate cancer patients in Shanghai Tenth People's Hospital. [file 5554708.f1.zip › 5554708.f1/Supplementary Table 1.docx]

**Supplementary Table 1** R packages used in this study.

| Statistical analysis | R package |
| --- | --- |
| mRMR | mRMRe |
| ROC | pROC |
| LASSO logistic regression | glmnet |
| SVM-RFE | e1071 |
| Logistic regression, nomogram, C-index, calibration plot | rms |
| Collinearity diagnosis | car |
| Hosmer-Lemeshow test | vcdExtra |
| DCA curve | ggDCA |
| NRI | nricens |
| IDI | PredictABEL |
