# Supplementary material for: A Genomic-Clinicopathologic Nomogram for the Prediction of Lymph Node Invasion in Prostate Cancer
Source: J Oncol. 2021 May 26;2021:5554708. doi: 10.1155/2021/5554708 (PMC8172299; doi:10.1155/2021/5554708)
Supplement: Supplementary Materials — Supplementary Figure 1: pairwise Spearman rank correlation among 37 selected genes of the support vector machine model in the SMOTE-balanced training set. Supplementary Table 1: R packages used in this study. Supplementary Table 2: the clinicopathological characteristics of five prostate cancer patients in Shanghai Tenth People's Hospital. [file 5554708.f1.zip › 5554708.f1/Supplementary Table 2.docx]

**Supplementary Table 2** The clinicopathological characteristics of five prostate cancer patients in Shanghai Tenth People’s Hospital.

| Characteristic | Age (year) | PSA level (before surgery), ng/mL | Clinical stage | Preoperative Gleason score | Prostatectomy Gleason score | pT stage | Positive surgical margins | Number of dissected lymph nodes | Number of positive lymph nodes |
| --- | --- | --- | --- | --- | --- | --- | --- | --- | --- |
| Patient 1 | 61 | 10.99 | cT2 | 8 (4 + 4; ISUP GG4) | 9 (4 + 5; ISUP GG5) | pT2c | No | 30 | 4 |
| Patient 2 | 79 | 7.17 | cT2 | 8 (4 + 4; ISUP GG4) | 9 (4 + 5; ISUP GG5) | pT3a | Yes | 25 | 3 |
| Patient 3 | 59 | 184.41 | cT3 | 9 (5 + 4; ISUP GG5) | 9 (5 + 4; ISUP GG5) | pT4 | Yes | 54 | 23 |
| Patient 4 | 71 | 32.22 | cT4 | 9 (4 + 5; ISUP GG5) | 9 (4 + 5; ISUP GG5) | pT4 | Yes | 46 | 35 |
| Patient 5 | 74 | 7.64 | cT2 | 7 (3 + 4; ISUP GG2) | 7 (3 + 4; ISUP GG2) | pT3a | No | 17 | 2 |

ISUP GG: International Society of Urological Pathology grade group

PSA: prostate-specific antigen

LNI: lymph node invasion
