# Supplementary material for: A Genomic-Clinicopathologic Nomogram for the Prediction of Lymph Node Invasion in Prostate Cancer
Source: J Oncol. 2021 May 26;2021:5554708. doi: 10.1155/2021/5554708 (PMC8172299; doi:10.1155/2021/5554708)
Supplement: Supplementary Materials — Supplementary Figure 1: pairwise Spearman rank correlation among 37 selected genes of the support vector machine model in the SMOTE-balanced training set. Supplementary Table 1: R packages used in this study. Supplementary Table 2: the clinicopathological characteristics of five prostate cancer patients in Shanghai Tenth People's Hospital. [file Supplementary Figure 1.pdf › 5554708.f1/Supplementary Figure 1.pdf]

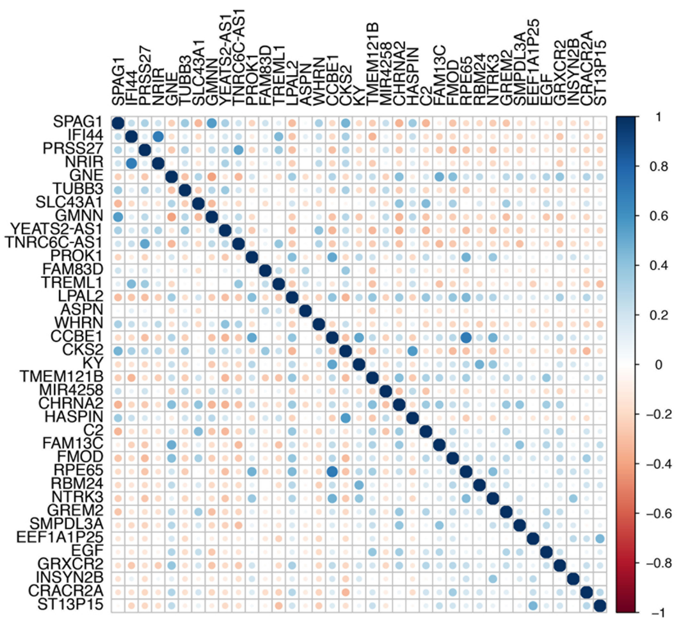

SPAG1
IFI44
PRSS27
NRIR
GNE
TUBB3
SLC43A1
GMNN
YEATS2-AS1
TNRC6C-AS1
PROK1
FAM83D
TREML1
LPAL2
ASPN
WHRN
CCBE1
CKS2
KY
TMEM121B
MIR4258
CHRNA2
HASPIN
C2
FAM13C
FMOD
RPE65
RBM24
NTRK3
GREM2
SMPDL3A
EEF1A1P25
EGF
GRXCR2
INSYN2B
CRACR2A
ST13P15
1
0.8
0.6
0.4
0.2
0
−0.2
−0.4
−0.6
−0.8
−1
